# Photothermal Properties of IR-780-Based Nanoparticles Depend on Nanocarrier Design: A Comparative Study on Synthetic Liposomes and Cell Membrane and Hybrid Biomimetic Vesicles

**DOI:** 10.3390/pharmaceutics15020444

**Published:** 2023-01-29

**Authors:** Júlia Muniz Barcelos, Tácio Gonçalves Hayasaki, Ricardo Costa de Santana, Eliana Martins Lima, Sebastião Antonio Mendanha, Andris Figueiroa Bakuzis

**Affiliations:** 1 Institute of Physics, Federal University of Goiás, Goiânia 74690-900, GO, Brazil; 2 Farmatec, School of Pharmacy, Federal University of Goiás, Goiânia 74690-631, GO, Brazil; 3 CNanomed, Federal University of Goiás, Goiânia 74690-631, GO, Brazil

**Keywords:** cancer, heat-triggered drug release, near-infrared dye, photoluminescence, doxorubicin

## Abstract

Biomimetic nanoparticles hold great promise for photonic-mediated nanomedicine due to the association of the biological functionality of the membrane with the physical/chemical goals of organic/inorganic structures, but studies involving fluorescent biomimetic vesicles are still scarce. The purpose of this article is to determine how photothermal therapy (PTT) with theranostic IR-780-based nanoparticles depends on the dye content, cholesterol content, lipid bilayer phase and cell membrane type. The photophysical responses of synthetic liposomes, cell membrane vesicles and hybrid nanoparticles are compared. The samples were characterized by nanoparticle tracking analysis, photoluminescence, electron spin resonance, and photothermal- and heat-mediated drug release experiments, among other techniques. The photothermal conversion efficiency (PCE) was determined using Roper’s method. All samples excited at 804 nm showed three fluorescence bands, two of them independent of the IR-780 content. Samples with a fluorescence band at around 850 nm showed photobleaching (PBL). Quenching was higher in cell membrane vesicles, while cholesterol inhibited quenching in synthetic liposomes with low dye content. PTT depended on the cell membrane and was more efficient for melanoma than erythrocyte vesicles. Synthetic liposomes containing cholesterol and a high amount of IR-780 presented superior performance in PTT experiments, with a 2.4-fold PCE increase in comparison with free IR-780, no PBL and the ability to heat-trigger doxorubicin release.

## 1. Introduction

In the last decades, several nanoparticles have been approved for clinical use, and lipid-based ones are among the most popular [1]. Examples span from lipid bilayer vesicles, for example, liposomes such as Doxil/Caelyx, which were approved in 1995/1996 and have been responsible for nearly 300 clinical trials so far, to new RNA-based vaccines against COVID-19, such as BNT162b2, that consist of complex lipid nanoparticles and received emergency use authorization by the FDA in 2020 [1]. Recently, a novel nanotechnology strategy emerged with a focus on associating biological membranes with organic/inorganic nanostructures to create biomimetic nanoparticles.

Cell-membrane-coated nanoparticles (CMNPs) are a new nanoplatform that unites the physical and chemical properties of the core nanoparticles (or therapeutic agents) with the biological function of a specific cell membrane coating. CMNPs may contain a chemotherapeutic molecule, an imaging contrast agent or tracer, or a photo- or magneto-responsive material, while cell membranes might have several biological functions, such as immune evasion, immune stimulation, antigen presentation or cancer targeting, among others [2]. Recent results indicate that such biomimetic systems can have great advantages in comparison to traditional pegylated nanotechnology [2]. For instance, erythrocyte-coated nanoparticles (NPs) enhance the blood circulation time in comparison with pegylated-NPs, even after several administrations [2,3]. In general, a longer blood circulation time results in a higher intratumoral delivery efficiency of the NP [2].

In addition, photo-mediated applications have received considerable attention due to potential clinical applications, including with CMNPs [4]. Most investigations that apply CMNPs for photothermal therapy (PTT) use inorganic nanoparticles, such as Au-based or iron oxide-based ones, while a few have used organic ICG as a PTT agent, probably due to limitations related to photobleaching of the NIR molecule [2].

Despite the great potential of the biomimetic approach, there are still several challenges for clinical translation. One of the greatest is the scaling-up process, which demands large bioreactors for the in vitro cell culture and aseptic conditions to guarantee the quality and assurance established by regulatory agencies [2]. It is expected that this approach will require large amounts of cell membranes for nanoparticle production. On the other hand, some well-known nanoparticles, such as synthetic lipid-based or iron oxide-based ones, are well established in the clinic, with several products on the market [1]. Synthetic nanoparticles can be produced on demand but might have enhanced performance if they are surface-modified, for example, by the hybridization of conventional liposome bilayers and cell membranes [5,6,7].

Furthermore, in contrast to CMNPs, there are not many reports on photonic-mediated applications using this bio-hybrid nanoparticle strategy. Similarly, there are few reports of synthetic lipids loaded with near-infrared organic photothermal agents, in particular, IR-780 molecules. Cationic IR-780 heptamethine dye is interesting due to its applications in NIR imaging, chemotherapy, photodynamic therapy (PDT) and PTT [8,9,10]. The photophysical properties of IR-780 are greater than those of clinically approved ICG; for instance, the fluorescence quantum yield is 10-fold higher, resulting in better cancer imaging and detection, which is useful for PDT due to the ability to produce singlet oxygen under 808 nm excitation and shows a better photothermal response [8,9]. IR-780 is a lipophilic molecule easily introduced into liposomes or cell-based nanoparticles [11,12,13,14,15,16,17,18]. Nevertheless, like several organic dyes, photobleaching can limit some photonic applications.

The purpose of this study was to investigate the role of several parameters in the photonic response of IR-780 when incorporated into synthetic liposomes, cell-based vesicles and hybrid liposomes. Our goal was to determine how the lipid bilayer phase, IR-780 amount and cell membrane type (erythrocytes or melanoma cells), among other parameters, tune the photophysical properties of the nanoplatform, aiming at enhanced biomedical applications. Several characterization techniques were used, namely, nanoparticle tracking analysis (NTA), zeta potential, UV/VIS/NIR spectrophotometry, photoluminescence (PL), electron spin resonance (ESR), and photothermal- and heat-triggered drug release experiments, among others. Doxorubicin (DOX) was chosen as a drug model due to its importance in chemo- and immunotherapy [19]. Although DOX and liposomes are widely investigated in the literature, only a few reports including IR-780 in nanocarriers have been published [13,16,17], while there are no reports on the use of IR-780 in hybrid vesicles. Even though more complex photo-mediated nanocarriers are being designed to enhance immunotherapy [18], the knowledge about the photophysical properties of such nanocarriers is still in its infancy. For example, there are no studies on the photothermal conversion efficiency of this type of nanocarrier, a property that indicates the percentage of electromagnetic energy that is transformed into heat by the interaction of non-ionizing radiation with the nanoparticle (in this case, IR-780).

In this study, we demonstrate that cholesterol inhibits the quenching of IR-780 fluorescence from lipids, and tuning the amount of IR-780 in the hybrid membrane inhibits photobleaching. We also observed that increasing the amount of IR-780 in the membrane results in a redshift of the fluorescence, suggesting a different arrangement of IR-780 molecules in the membrane. In contrast to other articles in the literature, we determined the photothermal conversion efficiency (PCE) of the nanocarriers using Roper´s method [20] and investigated the role of several membrane constituents. We found that the PCE decreases with higher laser power and a lower IR-780 concentration. The PCE values of the membrane-based nanoparticles are enhanced in comparison to free IR-780. Additionally, tuning the IR-780 concentration allows the enhancement of the nanocarrier’s photothermal properties. In the end, our best nanoparticle loaded with DOX was used to demonstrate in vitro heat-triggered drug release using the B16-F10 murine melanoma cell line as a model.

## 2. Materials and Methods

### 2.1. Chemicals

Soy phosphatidylcholine (PC, Lipoid S100) was purchased from Lipoid GmbH (Ludwigshafen, Germany); cholesterol (CHOL), 1,2-distearoyl-sn-glycero-3-phosphoethanolamine-N-[amino (polyethylene glycol)-2000] (DSPE-PEG) and IR-780 iodide were from Merck (Darmstadt, Germany); doxorubicin hydrochloride was from LCLabs (Woburn, MA, USA); and the spin label 5-doxyl-stearic acid (5-DSA) was purchased from Santa Cruz Biotechnologies (Dallas, TX, USA). Cell culture reagents such as Dulbecco’s Modified Eagle’s Medium, fetal bovine serum and penicillin–streptomycin were purchased from ThermoFisher Scientific (Waltham, MA, USA). All other reagents were purchased from Merck (Darmstadt, Germany) or ThermoFisher Scientific (Waltham, MA, USA) at the highest available purity grades.

### 2.2. Liposome Preparation

In this work, five different types of liposomes were used, namely, synthetic liposomes (SL) composed of PC or PC + 30 mol% CHOL, erythrocyte vesicles (ELs) and melanoma vesicles (MLs) composed of erythrocyte or B16-F10 cell membranes and, finally, two cell-membrane-derived hybrid vesicles (HELs and HMLs) composed of pre-formed SLs fused with membrane fragments derived from either ELs or MLs.

Synthetic liposomes (SLs) (25 mM) were prepared by dissolving soy phosphatidylcholine and cholesterol in chloroform to prepare a film of lipids by rotary evaporation under pressure (IKA RV 10). Subsequently, the films were hydrated using a 300 mM (NH_4_)_2_SO_4_(_aq_) buffer, pH = 3.0, to form multilamellar liposomal suspensions. Unilamellar liposomes were obtained after rigorous extrusion using polycarbonate filters with 0.1 μm diameter pores. Finally, the SL surface was modified by the addition of PEG-2000 molecules following a previously reported protocol [21]. Briefly, 5 mol% DSPE-PEG was dissolved in chloroform within a glass flask. The solvent was then forced to evaporate under a gaseous nitrogen flow, forming a thin PEG-lipid film. Next, SL liposomes were added to the film, and the final dispersion was subjected to slow orbital shaking (IKA, KS 400) for 12 h.

Doxorubicin (DOX) was encapsulated into SLs following a previously described method that involves both pH and [NH^4+^] gradients [21,22]. It is well known that these gradients favor DOX influx and entrapment within lipid vesicles by the formation of insoluble DOX-SO_4_^−2^ salt crystals in the aqueous core [22,23]. In this way, 2 mg/mL lyophilized DOX hydrochloride was added to the SL suspension, and the solution was kept at 4 °C for 12 h under slow orbital shaking (IKA, KS 400). The unentrapped DOX was removed by filtration, and the encapsulation efficiency was spectrophotometrically measured to be 90%.

Erythrocyte liposomes (ELs) were prepared using intact erythrocyte membranes isolated by hypotonic lysis following a previously described protocol [11]. Melanoma liposomes (MLs) were obtained after B16-F10 cell disruption by repeated freezing and thawing cycles followed by differential centrifugation for the isolation of membrane fragments, as described in Reference [24]. After erythrocyte/B16-F10 internal content removal and membrane isolation, the membrane suspensions (at ~1.5 mg of protein/mL) were rigorously extruded using polycarbonate filters with 0.1 μm diameter pores to form unilamellar cell membrane vesicles. Finally, hybrid erythrocyte liposomes (HELs)/hybrid melanoma liposomes (HMLs) were prepared by combining SLs and ELs or MLs in a 2:1 ratio (*w*/*w*). The HEL/HML mixture was subjected to 50 freeze and thaw cycles and then to further extrusion under pressure as described above to merge both SL and EL or ML membranes.

IR-780 molecules were incorporated into each studied liposome using a post-insertion protocol. A stock solution containing the dye diluted in chloroform was used to prepare thin films of different IR-780 concentrations at the bottom of glass tubes under a gaseous nitrogen flow. The liposome solutions were placed in contact with IR-780 films under slow orbital shaking (IKA, KS 400) for 30 min, and the excess dye was removed by filtration.

### 2.3. Liposome Characterization

Liposomes were characterized in terms of their size, zeta potential, concentration, membrane fluidity, morphology and IR-780 content. For HELs and HMLs, the total protein content was also determined. The liposome size and concentration were evaluated using a nano-tracking analysis device (NanoSight NS500, NanoSight, Amesbury, UK) equipped with a 532 nm laser and an EMCCD 215S camera. The vesicle suspensions were diluted in a ratio of 1:100,000 before analysis and automatically injected into the sample compartment. Final sample concentrations and size distributions were obtained using the NTA 3.4 software (NanoSight, Amesbury, UK). Subsequently, liposomes’ zeta potentials were determined using a NanoBrook Zetaplus (Brookhaven, GA, USA). The membrane fluidity of spin-labeled vesicle suspensions was evaluated by EPR measurements using a Bruker EMX Plus spectrometer (Rheinstetten, Germany) operating in the X-band (approximately 9.4 GHz) with a 4119-HS resonant cavity and the following instrumental parameters: microwave power, 2 mW; modulation frequency, 100 kHz; amplitude of modulation, 1 G; magnetic field scan, 100 G; scan time, 168 s; and detection time constant, 41 ms. The maximum hyperfine splitting parameter (2A_||_) values were obtained from the experimental spectra and were used as a measure of membrane rigidity, as previously described [25]. Liposome morphologies were assessed by transmission electron microscopy (TEM). The vesicle suspensions were fixed using a buffered formaldehyde solution (25%, pH = 7.0) and post-fixed with an osmium tetroxide 4% solution and subsequently dehydrated in ethanol. Finally, the samples were deposited on carbon films of a TEM copper grid and colored by 0.5% aqueous uranyl acetate. The images were acquired using a JEOL JEM-2100 microscope (Tokyo, Japan). The IR-780 content was determined using a Cary 50 UV-Vis spectrophotometer (Varian Inc., Palo Alto, CA, USA) using a calibration curve of known IR-780 concentrations. HEL and HML total protein content was determined using a commercial kit (Sigma-Aldrich, Burlington, MA, USA) based on the reaction of bicinchoninic acid (BCA) to confirm membrane fusion. Briefly, HEL/HML samples were added to a solution of the BCA reagent, and after incubation for 30 min at 37 °C, the absorbance was measured at 562 nm. The protein concentration was determined using a calibration curve prepared with known concentrations of bovine serum albumin.

### 2.4. Cell Culture and Viability Assays

B16-F10 murine melanoma cells were grown in DMEM (Dulbecco’s Modified Eagle’s Medium) supplemented with 10% FBS (fetal bovine serum) and 1% penicillin–streptomycin in a 5% CO_2_ atmosphere at 37 °C. Before viability assays, cells were allowed to grow for 24 h in 96-well plates. Subsequently, cells were exposed to the studied liposome suspensions for an additional 24 h prior to MTT viability assays.

### 2.5. Photophysical Properties (Absorption and Photoluminescence)

The absorption curves were recorded in the 200–1000 nm wavelength range at room temperature using a Cary 50 UV-Vis spectrophotometer (Varian Inc., Palo Alto, CA, USA) equipped with a full-spectrum Xe pulse lamp single source. All liposome samples were diluted to prevent light scattering. Emission measurements were performed in a Horiba-Jobin Yvon spectrofluorimeter (Tokyo, Japan), Model Fluorolog-3 (FL3-221), under excitation with an external laser source of 804 nm (80 mW) connected to a Spectracq2 data acquisition module and an R5509-73 PMT InGaAs detector for IR measurements. All spectra were recorded for liquid samples in quartz cuvettes with 1 mm optical length and at room temperature.

### 2.6. Near-Infrared Imaging

Fluorescence molecular tomography (FMT) was performed using an upgraded FMT 1500 Fluorescence Tomography in vivo Imaging System bought from PerkinElmer (Waltham, MA, USA), which operates at 4 channels, namely, 635, 680, 750 and 790 nm. The fluorescence study used the 790 nm excitation channel with a maximum laser output power of 80 mW. Samples were measured in 2D fluorescence mode.

### 2.7. Photothermal Experiments

The photothermal therapy (PTT) experiments used a diode laser (model Laser iZi 808) bought from LASERline (Sao Paulo, Brazil), with an 808 nm wavelength. The sample holder contained 150 μL of the nanocarrier. The photothermal properties of the nanocarrier were investigated at 3 distinct laser powers, namely, 100, 200 and 300 mW. The temperature was monitored using an infrared thermal camera bought from FLIR, model SC 620 (Wilsonville, OR, USA). The same ROI centered in the laser spot of the sample was used to report the mean temperature during PTT. The thermal procedure consisted of monitoring the temperature with the laser off for 30 s, and then we turned on the laser until the stationary regime was achieved. When the sample did not show photobleaching, heating was maintained for 40 min; otherwise, a shorter heating time was considered. After a few minutes in the stationary regime, we turned off the laser and monitored the cooling process for more than 20 min.

The in vitro heat-triggered antitumoral activity of SLs containing DOX was investigated by irradiating a cell culture using the same laser. B16-F10 cells (96-well plate, 10,000 cells/well) were allowed to grow for 24 h and then exposed to liposomes for 30 min, followed by laser irradiation to achieve the desired thermal dose or the maximum dose in the high-power condition. The laser irradiation time was 5 min for temperatures close to 43–44 °C or 5 min for samples that did not achieve significant temperature variation. Temperatures close to 43–44 °C were controlled by tuning the laser power. The maximum laser power used was 300 mW for the control (cells without nanoparticles). Finally, the cells were incubated in a 5% CO_2_ atmosphere at 37 °C for 24 h prior to MTT viability assays.

### 2.8. Photothermal Conversion Efficiency Determination

The percentage of electromagnetic energy that is converted into heat is named the photothermal conversion efficiency (PCE). To obtain PCE values for all samples, we used Roper´s method during the cooling process [20]. Basically, the PCE value η is obtained from the equation:

(1) $$\eta =\frac{hS\left(T_{max}-T_{env}\right)-\dot{Q_{0}}}{P\left(1-10^{-A_{\lambda }}\right)}$$

where $T_{max}$ and $T_{env}$ are the stationary (maximum) and environmental temperatures, respectively, ${\dot{Q}}_{0}$ is the nonspecific power absorbed by the sample holder and liquid carrier, P is the laser power, $A_{\lambda }$ the sample absorption at wavelength λ, h is the heat transfer coefficient, and S is the surface area irradiated during PTT. All of these terms can be obtained experimentally, as discussed by Roper [20], except the term hS, which is determined after the analysis of the cooling profile (for details, please check the SI of Ref. [26]). Defining a dimensionless temperature parameter as:

(2) $$\Theta =\frac{T_{env}-T\left(t\right)}{T_{env}-T_{max}}$$

and solving the power balance equation for a sample irradiated with a laser power P and wavelength λ, it is possible to show that the solution during the cooling is:

(3) $$\Theta =e^{-t/\tau_{s}}$$

where t is the time after turning off the laser, while $\tau_{s}$ is the thermal relaxation time, given by:

(4) $$\tau_{s}=\frac{\sum_{i}m_{i}c_{p,i}}{hS}$$

Note that the sum is over all of the materials involved, which means the sample holder, the liquid carrier and the nanocarrier (nanoparticle). $m_{i}$ and $c_{p,i}$ are the mass and heat capacity of material *i*, respectively. In summary, fitting the cooling curve makes it possible to determine the thermal relaxation time, which, together with other parameters previously determined experimentally, allows us to calculate the photothermal conversion efficiency (η) value. Unfortunately, our experimental data during the cooling regime are not perfectly fitted with only one exponential. To estimate the PCE, only the region with 0.5 < −lnθ < 1.5 is considered. Data from three distinct regions are used for the fit and extraction of the thermal relaxation time: (i) 0.5 < −lnθ < 1.0, (ii) 1.0 < −lnθ < 1.5 and (iii) 0.5 < −lnθ < 1.5. The error of the PCE is the standard deviation of the photothermal conversion efficiency values obtained from the theoretical analysis.

### 2.9. Statistical Analysis

Statistical data analyses were performed via analysis of variance (ANOVA) and/or Student’s *t*-test using OriginLab 8.5 (Northampton, MA, USA) and GraphPad Prism 9.0 (San Diego, CA, USA) software. Statistically significant differences were considered for *p* < 0.05.

## 3. Results

### 3.1. Nanoparticle Size Distribution and Zeta Potential

**Figure 1:**
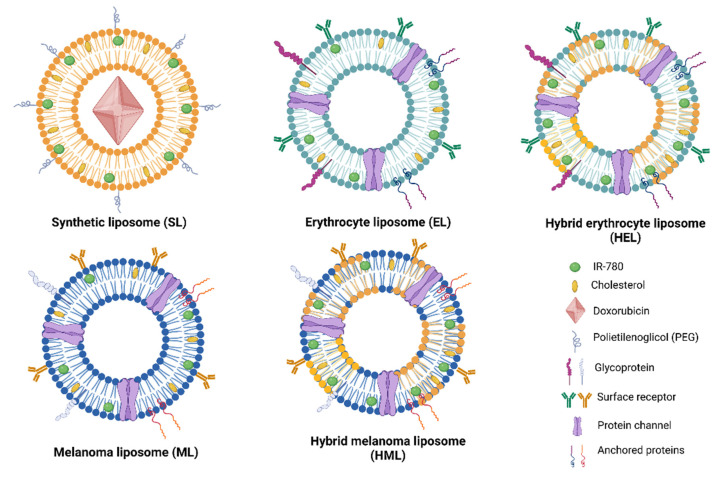
Schematic figure showing the five types of nanoparticles investigated, namely, synthetic liposomes (SLs); cell-membrane-based nanostructures, erythrocyte (ELs) and melanoma B16F10 vesicles (MLs); and hybrid liposomes formed from the fusion of synthetic lipids and cell membranes, hybrid erythrocyte liposomes (HELs) and hybrid melanoma liposomes (HMLs).

Figure 1 shows the distinct type of vesicles investigated in this article, namely, synthetic liposomes (SLs), erythrocyte vesicles (ELs), melanoma vesicles (MLs), hybrid erythrocyte liposomes (HELs) and hybrid melanoma liposomes (HMLs). SLs had their surfaces modified by the addition of polyethylene glycol (PEG). In addition, the photothermal agent IR-780 was incorporated in different concentrations into the lipid vesicle bilayer, while doxorubicin (DOX) was encapsulated in SLs.

Figure S1 shows the size distribution obtained using nanoparticle tracking analysis (NTA) for all samples investigated. We analyzed different types of synthetic liposomes having the following components: only phosphatidylcholine lipids (PC) and PC with the addition of 30 mol% or 40 mol% cholesterol. We found that all synthetic liposomes have similar sizes regardless of their constituents (cholesterol, PEG and IR-780), although slight differences were found. The diameter of the synthetic liposomes containing PEG and IR-780 was independent of the cholesterol content, with a mean size close to 120 nm, while for the cell membrane vesicles, we obtained 178 nm for erythrocyte vesicles (ELs) and 198 nm for melanoma liposomes (MLs). The incorporation of doxorubicin in the synthetic liposome with 40 mol% cholesterol increased its mean size to 164 nm. The hybrid liposomes showed larger mean sizes: 197 nm for HELs and 232 nm for HMLs. Both hybrid and cell membrane nanoparticles showed broader size distributions.

Figure S2 shows the zeta potentials for all samples. We found that the incorporation of the lipophilic molecules IR-780 and cholesterol did not influence the nanoparticles’ surface charge. The synthetic liposome containing PC, IR-780 and cholesterol had a zeta potential of −8 to −12 mV, while SL-DOX changed its zeta potential to −22 mV. On the other hand, the mean zeta potential changed considerably for the cell membrane nanoparticles and the hybrid liposomes. We found −24 mV for ELs and −14 mV for MLs, while for HELs and HMLs, we obtained −26 mV and −6 mV, respectively. Higher standard deviations for cell and hybrid liposomes in comparison with the synthetic nanoparticles were found. Table 1 summarizes the physical characteristics of the samples investigated.

### 3.2. IR-780-Based Nanoparticles’ Optical Properties

**Figure 2:**
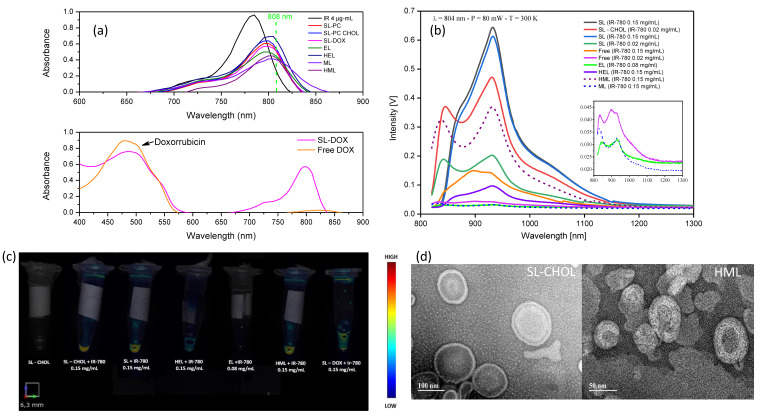
Photophysical and morphological properties of the nanoparticles: (**a**) UV-VIS-NIR absorption spectra of free IR-780, free DOX, SLs, SL-CHOL, ELs, MLs, HELs and HMLs; (**b**) fluorescence spectra of free IR-780, SLs, SL-CHOL, ELs, MLs, HELs and HMLs after excitation at 804 nm. Samples with different amounts of IR-780 are shown. The inset shows the spectra of free IR-780 0.02 mg/mL, ELs and MLs, confirming the band at around 850 nm; (**c**) fluorescence molecular tomography images of SL-CHOL without IR-780 (control sample), SLs, SL-CHOL, ELs, HELs, HMLs and SL-CHOL with DOX. (**d**) TEM images of SL-CHOL and HMLs.

Figure 2a shows the absorption spectra of several samples: free IR-780 dispersed in ethanol, synthetic liposomes with IR-780 (with and without cholesterol), membrane-based vesicles (erythrocyte and melanoma) and hybrid nanoparticles obtained from the fusion of cell membranes with synthetic lipids. The maximum absorption is observed for the dye at 784 nm, followed by synthetic liposomes (798 nm), hybrid liposomes (801 nm) and cell membrane vesicles (804 nm). It is clear from the data that the incorporation of IR-780 in the vesicles results in a redshift and a decrease of the maximum absorption, especially for the cell membrane vesicles. The dashed line represents the excitation wavelength and shows that absorption is higher for the SLs in this condition, followed by the hybrid vesicles, with similar values for free IR-780 and the cell membrane vesicles. SL-DOX showed the characteristic peak of doxorubicin close to 500 nm, confirming the encapsulation of DOX in the vesicle.

Figure 2b shows the photoluminescence (PL) of the same samples excited at 804 nm. The PL intensity is higher for the synthetic liposomes, decreasing for free IR-780, hybrid liposomes and cell membrane vesicles, in that order. Lower PL intensities were found for free IR-780 0.02 mg/mL, ELs and MLs, as shown in the inset. The PL intensity increases with increasing IR-780 content, and the spectra show three bands. For the SLs, two band positions, the main peak at around 932 nm and a shoulder peak at 1035 nm, are not influenced by the IR-780 content, while the other one shows a redshift when increasing the dye content in the membrane, shifting from 846 nm to 874 nm. Cholesterol seems to play an important role in photoluminescence at a low concentration of IR-780 nm. In this case, the PL showed around a 2-fold increase for synthetic liposomes containing cholesterol, suggesting that it inhibits quenching. On the contrary, hybrid vesicles showed a huge decrease in PL in comparison to free IR-780 and the SL. This result suggests that proteins on the cell membrane might be related to this quenching effect.

Figure 2c shows the 2D fluorescence images of the liposome samples obtained using FMT with excitation at 790 nm. Obviously, only samples containing IR-780 show fluorescence. This result indicates that from the samples prepared, the synthetic liposomes and the hybrid vesicles are the most relevant for near-infrared imaging. HMLs show a better FMT image than HELs. The cell membrane vesicle (EL) also shows fluorescence but at a lower intensity in comparison with the other samples, in accordance with the PL data reported in Figure 2b. In Figure 2d, one can see the morphological features of the synthetic (SL-CHOL) and hybrid (HML) liposomes. In both images, the lipid vesicle aspect is present, while amorphous membrane fragments are not detected, indicating the integrity of SL-CHOL liposomes and the success of HML hybridization.

### 3.3. Photothermal Therapy Potential of IR-780-Based Liposomes

#### 3.3.1. The Role of Cholesterol and IR-780 in PTT

**Figure 3:**
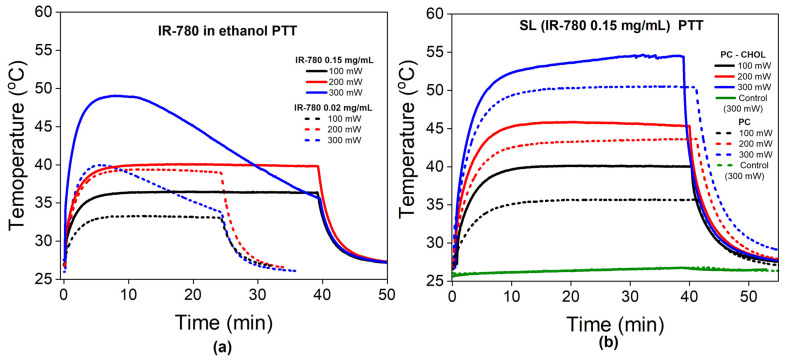
Photothermal study: (**a**) Temperature profile during PTT with free IR-780 in different amounts (0.02 and 0.15 mg/mL) and at different laser powers (100, 200 and 300 mW). Solid and dashed lines correspond to high and low IR-780 contents, respectively. (**b**) Temperature profile during PTT for synthetic liposomes containing a concentration of IR-780 of 0.15 mg/mL. Dashed lines represent SLs without cholesterol, while solid ones represent those with cholesterol.

Figure 3 shows the temperature profile during the photothermal experiment with free IR-780 (a) and synthetic liposomes (b), with excitation at 808 nm and different laser power conditions.

Figure 3a shows the temperature as a function of time during PTT for free IR-780 dispersed in ethanol at low and high concentrations of 0.02 mg/mL (dashed lines) and 0.15 mg/mL (solid lines), respectively. The temperature increases with increasing laser power and IR-780 content, as expected. However, at the highest laser power (300 mW), independent of the concentration of the dye, we observe the photobleaching phenomenon. In this experimental condition, therapeutic temperatures (defined as higher than 43 °C) are only achieved for the highest concentration, and thermal dose control is not possible due to photobleaching.

Figure 3b shows the PTT experiments for synthetic liposomes with 0.15 mg/mL IR-780 at different laser powers. Samples with cholesterol are indicated by solid lines, while those without cholesterol are represented as dashed lines. The water sample control at the highest power (300 mW) does not show a significant temperature rise during PTT, while all SL samples show an increase in temperature. Therapeutic temperatures are observed at lower laser powers for several samples (in comparison with free IR-780). More importantly, the delivered thermal dose can be tuned since there is no photobleaching under these conditions, which is a necessary property for several clinical applications. However, SL samples with 0.02 mg/mL showed photobleaching in all experimental conditions (see Figure S3 and Table S1). In addition, we found that samples with cholesterol achieve higher temperatures in the stationary regime in comparison with SL samples without cholesterol in their composition.

#### 3.3.2. The Cell Membrane Influences PTT

**Figure 4:**
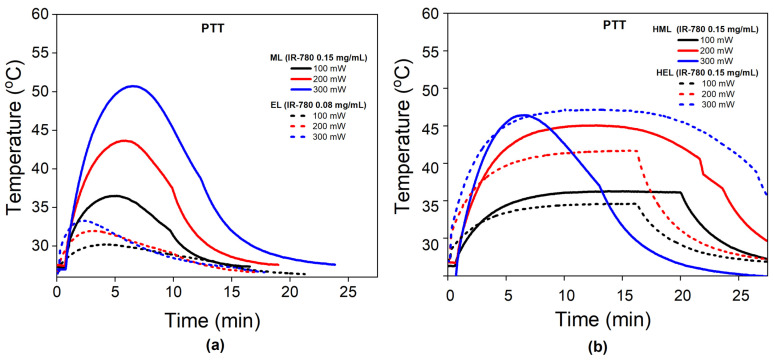
The influence of membrane type on PTT: (**a**) Temperature profile during PTT for cell membrane nanoparticles at different laser powers (100, 200 and 300 mW). Dashed lines represent erythrocyte vesicles (ELs), while solid ones indicate melanoma liposomes (MLs). (**b**) Temperature profile during PTT for hybrid nanoparticles at different laser powers (100, 200 and 300 mW). Dashed lines represent hybrid erythrocyte vesicles (HELs), while solid ones indicate hybrid melanoma liposomes (HMLs).

Figure 4a shows the temperature profile during PTT for the different cell membrane vesicles (erythrocyte and melanoma) at different laser power settings. Increasing the power enhances the heat generation, as expected. All EL samples show photobleaching, although at distinct times of irradiation, and none achieved therapeutic temperatures. On the other hand, the melanoma vesicles achieve therapeutic temperatures but also show photobleaching. Curiously, different from the EL, the time of irradiation before observing photobleaching increases with increasing laser power.

Figure 4b shows the PTT data for the hybrid liposomes, HELs and HMLs. The hybrid liposomes achieve (in general) higher temperatures than the cell membrane vesicles, indicating better photothermal performance. However, both samples show photobleaching, where the hybrid erythrocyte liposome shows it at 300 mW, while the hybrid melanoma liposome shows it at 200 mW. Both HELs and HMLs show photobleaching after achieving at least 45 degrees Celsius. This result is different from the cell membrane nanoparticles, which show the same phenomenon at lower temperatures.

**Figure 5:**
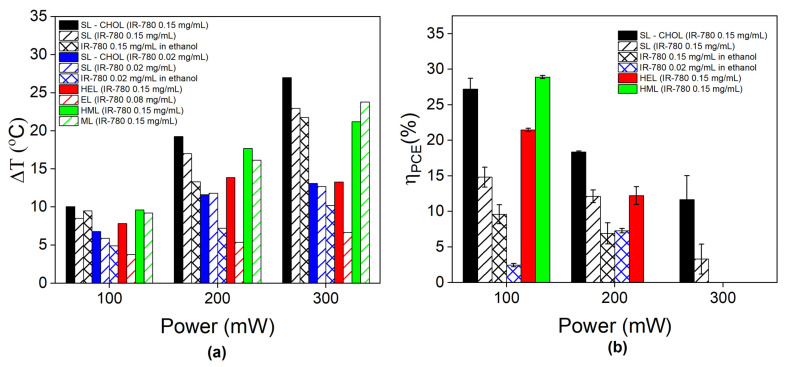
Photothermal efficiency: (**a**) Maximum temperature variation during PTT at different laser powers (100, 200 and 300 mW) for free IR-780, SLs, SL-CHOL, ELs, MLs, HELs and HMLs. A few samples show data for different amounts of IR-780. (**b**) Photothermal conversion efficiency (PCE) calculated using Roper´s method in different laser power conditions for free IR-780, SLs, SL-CHOL, HELs, and HMLs. Samples that show photobleaching were not considered in PCE calculations.

Figure 5a shows the temperature variation (maximum minus room temperature) for all samples and experimental conditions, while Figure S3 shows the maximum temperatures achieved. Therapeutic temperatures are only achieved at a power greater than 100 mW. Most of the samples show an increase in temperature variation with increasing power. However, free IR-780 and synthetic liposomes with low dye content (0.02 mg/mL) and containing cholesterol show saturation in high-power conditions. The same phenomenon is observed for the erythrocyte hybrid vesicle. On the contrary, the temperature variation increases with increasing power for several other samples with 0.15 mg/mL, such as SL-CHOL, SLs, MLs, HMLs and free IR-780.

#### 3.3.3. The Photothermal Conversion Efficiency Depends on the Constituents of the Vesicle

Figure 5b shows the photothermal conversion efficiency (PCE) calculated using Roper’s method during the cooling process. The standard deviation arises from curve fitting the cooling process at different time periods, as discussed later. Samples that show photobleaching are not considered. The value of the photothermal conversion efficiency decreases with increasing laser power. Higher PCE values are found for synthetic liposomes containing cholesterol (SL-CHOL), followed by hybrid liposomes, SLs without cholesterol content (SLs) and free IR-780, in that order. For instance, at 200 mW, we found a PCE of 17% for SL-CHOL, followed by HELs and SLs at around 12% and free IR with a value close to 7%. Different from the 100 mW condition, the IR-780 content had no influence on the PCE for free molecules suspended in ethanol. At 300 mW, the SL with cholesterol shows a PCE of 11%, while for the sample without cholesterol, it decreases to 3%.

#### 3.3.4. Cell Viability Study without NIR Irradiation

**Figure 6:**
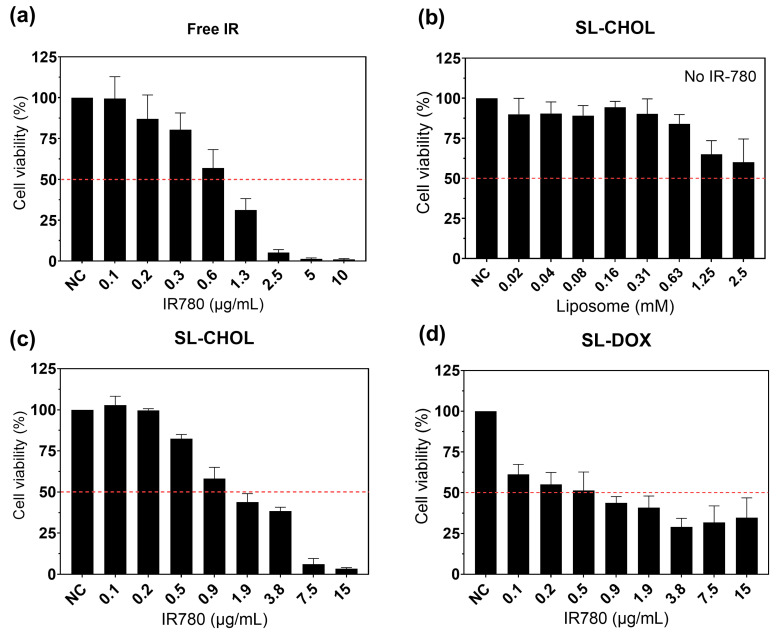
Cell viability study without laser irradiation: (**a**) MTT study as a function of free IR-780 concentration; (**b**) MTT investigation of the toxicity of SL-CHOL without IR-780; (**c**) MTT study of SL-CHOL containing IR-780; (**d**) MTT study of SL-DOX. DOX encapsulated in the vesicles decreases the IC50. The variation in concentration (IR-780) arises from the dilution of the original sample.

Figure 6 shows the cell viability studies with free IR-780 (Figure 6a), SL-CHOL without IR-780 (Figure 6b), SL-CHOL with IR-780 (Figure 6c) and SL-DOX (Figure 6d). The dashed line represents the IC50 for each study. We found that IR-780 has a chemotherapeutic effect even without irradiation, with an IC50 close to 1 μg/mL. The SL-CHOL sample without IR-780 showed no toxicity in a large liposome concentration range. On the other hand, SL-CHOL with IR-780 showed a slightly higher IC50 in comparison to free IR-780, around 1.4 μg/mL. In addition, the incorporation of DOX in the SL-CHOL sample resulted in a decrease in IC50 to 0.47 μg/mL due to both chemotherapeutic agents, i.e., IR-780 and doxorubicin. The DOX content in the liposome is responsible for the greater chemotherapeutic efficiency of SL-DOX (see Figure S4 for the viability results as a function of liposome concentration).

#### 3.3.5. Heat-Triggered Doxorubicin Release

**Figure 7:**
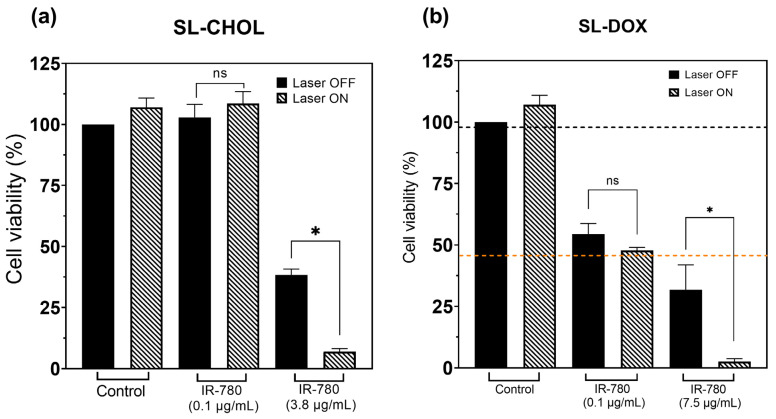
Photothermal effect on cell viability: (**a**) MTT data of SL-CHOL with and without laser irradiation (PTT); (**b**) MTT data of SL-DOX with and without PTT. The orange and black horizontal lines in (**b**) indicate the cell viabilities of free DOX at 7.5 μg/mL (37 °C) and a control sample in a thermal bath experiment maintained at 43 °C for 4 h [21], respectively. The temperature profiles during PTT of representative samples for both figures (**a**) and (**b**) are shown in the Supplementary Material. The highest thermal dose is achieved with SL-DOX containing 7.5 μg/mL IR-780. * *p* < 0.05 (*t*-test).

Figure 7a shows the cell viability results of SL-CHOL containing IR-780 with and without near-infrared laser irradiation. Cells without nanoparticles irradiated at the highest laser power (300 mW) do not show any significant temperature variation (see Figure S5). At a low IR-780 concentration, the cells also do not heat. On the contrary, SL-CHOL with 3.75 μg/mL shows an effect after PTT. The data show that increasing the IR-780 content results in a decrease in cell viability, and that if the amount of IR-780 is enough to generate heat, one also observes a decrease in viability. For the largest IR-780 concentration, the viability changed from 38% to 7% after PTT.

Figure 7b shows the heat-triggered doxorubicin release study of SL-DOX samples. Cell viability is not affected by PTT without nanoparticles, where the laser alone is not able to increase the temperature of the cell liquid media (see Figure S5). Without laser irradiation, due to doxorubicin, even a concentration of 0.1 μg/mL results in a decrease in cell viability to 54%. Irradiation slightly changes the viability to 48%, but without any statistical significance. The medium containing the cells does not show a significant increase in temperature (see Figure S5). However, increasing the concentration of IR-780 and DOX results in a decrease in cell viability to 32% due to the higher concentration. More importantly, during PTT, the cells achieve temperatures on the order of 44 degrees, as shown in Figure S5. For this sample, we calculated the CEM43 to be 6.5 min. So, at the highest DOX and IR-780 concentrations, the viability changed from 32% to 3% after PTT.

## 4. Discussion

Several studies have used synthetic liposomes enriched with IR-780 and therapeutic agents (chemotherapeutical, phytopharmaceutical and PDL1 inhibitors, among others) [12,13,14,15,16,17,18]. On the other hand, cell membrane nanoparticles containing IR-780 have been reported only recently, while so far, no reports are found for hybrid liposomes complexed with IR-780 [2,11]. For instance, our group previously coated Mn-doped iron oxide-based nanoparticles with red blood cell membranes containing lipophilic IR-780 molecules. The nanocarrier showed a long blood circulation time and has the potential for NIR, MRI and alternating current biosusceptometry (ACB) imaging and thermal nanomedicine therapeutics (magnetic hyperthermia and photothermal therapy) [11].

Furthermore, few studies have examined CMNP fusion with different cell membranes. Wang et al. fused RBCs and B16-F10 melanoma cells for the encapsulation of DOX and hollow copper sulfide (CuS) NPs [27]. The hybrid CMNPs showed a long blood circulation time, higher intratumoral delivery efficiency than single cell membranes and applications for PTT-mediated drug release. Xiong et al. fused RBCs and ID8 ovarian cancer membranes with iron oxide and ICG. The cell membranes of ID8 stimulated immune responses, but those of B16-F10 did not. This strategy indicates synergistic PTT–immunotherapy for ovarian cancer [28]. Most articles exploring PTT generate heat with ICG molecules and/or inorganic nanoparticles, such as gold, iron oxide or copper sulfide [2,27,28]. Sun et al. developed doxorubicin (DOX)-loaded gold nanocages coated with 4T1 cell membranes for cancer targeting and heat-triggered drug release under near-infrared (NIR) irradiation [29]. Bahmani incorporated ICG dyes in erythrocyte-membrane-derived nanoparticles for imaging and PTT [30]. Here, we explored in detail the potential of IR-780-based nanoparticles and investigated the role of several factors, such as the dye content, cholesterol, lipid bilayer phase and cell membrane type, among other factors.

The distinct liposome-based nanoparticles prepared containing the photothermal agent IR-780 were synthetic liposomes, cell membrane vesicles (melanoma cells and erythrocytes) and hybrid liposomes (fusion of cell membranes and synthetic lipids). A schematic representation of the samples is shown in Figure 1. We found that the mean size of the synthetic liposomes was close to 120 nm, while the hybrid and cell membrane vesicles ranged from 180 to 230 nm, respectively, presenting larger dispersity. A rigorous extrusion process was used for sample preparation, but we noticed that to obtain 100 nm cell membrane vesicles and hybrid liposome suspensions, several additional steps using membranes with pores of 100–200 nm were required. As a result, we verified a higher loss in the sample’s lipid/protein content and a drop of an order of magnitude in the particle concentration (see Table 1). So, to manage the particle concentration, we decided to limit the extrusion cycles and use 200 nm pore-size membranes to extrude the membrane-derived vesicles. Consequently, these samples were characterized by inhomogeneous size distributions and higher mean sizes. These results suggest that the membrane-protein content may be a limiting factor in the preparation of hybrid liposomes with a reduced mean size, at least for suspensions with a high particle concentration. In fact, Ferrel et al. [7] fused erythrocyte membranes and synthetic liposomes to obtain a relatively narrow size distribution curve after 200 nm pore extrusion. However, they reported suspensions containing approximately 1 mg/mL lipids/erythrocyte membranes, while in this work, we used almost 10 mg/mL. Nevertheless, the final liposome suspension’s lipid/protein concentration may always be tuned to fulfill its desired function.

So far, there is only one near-infrared imaging agent approved for clinical use, ICG, but a few others are under investigation [9]. IR-780 was chosen because of its multifunctionality, i.e., better imaging properties and PTT and PDT applications [8,9], and also because of its lipophilic character, which guarantees the incorporation of the dye in the membrane [11,12,13,14,15,16,17,18].

We observed that the optical properties changed when introducing IR-780 into the membrane; it depended on the cholesterol content, dye concentration and protein amount (see Figure 2). This agrees with computer simulations that clearly demonstrate that distinct arrangements inside the lipid bilayer result in changes in the optical properties of fluorescent probes [31]. Our results show that fluorescent intensity is lower for free IR-780 than for synthetic liposomes. This is easily explained by the redshift of the absorbance when incorporated in the membrane: it is higher for the liposomes in comparison with free IR-780 at 808 nm excitation (see Figure 2a). We also observed that for low IR-780 content, photoluminescence is higher when cholesterol is incorporated in the liposome lipid bilayer. Furthermore, the redshift of the low peak band at the higher IR-780 amount also suggests that the fluorescent probes are arranged differently in the bilayer. This indicates that the photophysical properties of IR-780 incorporated in liposome membranes are modulated by cholesterol in a concentration-dependent manner. It is well known that cholesterol at adequate molar fractions induces a lipid bilayer reorganization and enhances the membrane rigidity of different lipid mixtures [32,33], and this is also verified in this work by ESR experiments (see Table 1 and Figure S6). Our results suggest that for IR-780 at a low concentration, enhanced lipid rigidity due to the cholesterol presence and consequently the local IR-780 microenvironment modification can inhibit quenching by the lipids and/or from solvent infiltration. In addition, the decrease in lateral molecular diffusion might prevent IR-780 clustering in the membrane. However, is worth noting that the presence of IR-780 also promoted membrane rigidity, as indicated by the increase in the ESR-motional parameter 2A_||_values of all samples containing the dye at 0.15 mg/mL. On the other hand, the cell membrane vesicles clearly show a significant decrease in fluorescence. This could be happening due to quenching arising from the proteins or the coupling of IR-780 in the hydrophobic sites of some membrane proteins, which inhibits fluorescence. A previous study indicated that IR-780 interacts strongly with BSA proteins [10]. It will be extremely interesting in the future if computer simulations are used to improve our understanding of how the molecules are arranged in the lipid bilayer under such different conditions.

Photobleaching (PBL) was observed for free IR-780, cell membrane vesicles, hybrid liposomes and synthetic liposomes with the lowest IR-780 content (0.02 mg/mL), but not for SLs with 0.15 mg/mL IR-780 under similar experimental conditions. PBL arises due to the energy transfer from the probe’s triplet excited state to nearby molecular oxygen [34], which might generate reactive oxygen species (ROS). On the one hand, chemical reactions with ROS are attractive because they can promote rapid cell death, but on the other, they might limit the delivery of specific thermal doses that demand the control of the temperature for a certain treatment duration. The PBL phenomenon is expected to be enhanced with increasing laser power and temperature. Several samples show this feature; for example, free IR-780, HELs and HMLs only show PBL at higher laser power settings, while SLs (with low IR-780 content), MLs and ELs show it at all power settings. Indeed, cell membrane vesicles show PBL at lower laser power than free IR-780 or hybrid liposomes, indicating that the chemical reaction with ROS is more easily achieved by those types of nanoparticles.

In general, we observed that PBL happens after a shorter irradiation time with increasing laser power; the exception is the melanoma vesicle, which showed the opposite trend (see Figure 4a). It is not clear yet exactly why this happened with the ML, but the data suggest that surface proteins and the arrangement of IR-780 in the lipid bilayers containing them are important. Indeed, the ESR data in Table 1 show that the melanoma membrane fluidity is far higher than that of the erythrocyte membrane. This suggests that diffusion phenomena in the ML could be related to the increase in irradiation time for PBL in this type of cell since the temperature, cholesterol content and type of fatty acids influence fluidity. No difference is observed for hybrid liposomes containing erythrocyte or melanoma membranes, probably because the final lipid bilayer mobility was dominated by their SL portion, as demonstrated by ESR-2A_||_ parameter values, essentially, 52.0 G (see Table 1). Overall, the results indicate that this type of organic dye and cell membrane vesicles might have limited applications for PTT. For such nanostructures, the envisioned PTT application could be improved with inorganic photo-responsive materials [2,10,11].

It might be interesting to mention that samples showing a photoluminescence peak at around 850 nm presented photobleaching, suggesting that this peak position is related to some specific IR-780 arrangement. Increasing the concentration of IR-780 in the liposomes causes the redshift of the peak position and enhances PL at the 930 nm peak (see Figure 3b), which could be related to a distinct configuration of IR-780 in the lipid bilayer, therefore inhibiting photobleaching. From the experimental data, we conclude that SL samples with high contents of IR-780 and cholesterol are the best for PTT applications since they inhibit PBL, and one can easily tune the thermal dose (temperature and time) according to clinical necessity. However, it might be fair to mention that the dye configuration in the lipid bilayer might involve several parameters, and increasing the concentration might not always prevent certain dye arrangements that could result in PBL. Nevertheless, our data indicate that photoluminescence could be relevant for monitoring such a possibility.

Therapeutic temperatures (higher than 43 °C) were achieved by a few samples at a laser power of 200 mW (see Figure S5). The highest maximum temperature was achieved by SL-CHOL at 46 degrees, followed by HMLs at 45.4 °C, SLs at 43.7 °C and MLs at 43.6 °C, but HMLs and MLs showed PBL. The lowest temperature was achieved by the EL sample not only because of the lower IR-780 content (0.06 mg/mL instead of 0.15 mg/mL) but also because of the characteristics of the erythrocyte membrane. Note that free IR-780 at 0.02 mg/mL achieved higher temperatures than ELs (see Figure S5 and Table S1).

Similar conclusions are corroborated by evaluating the photothermal conversion efficiency (PCE) values. Note that, in this case, error bars arise due to the standard deviation in the estimation of PCE. We notice that the entire cooling regime of our samples is not perfectly fitted with only one exponential. This is probably due to distinct thermal relaxations from the sample. To better estimate PCE, we divided the cooling period into three distinct regions and estimated the thermal relaxation in each one. The error is the standard deviation obtained after the theoretical analysis. Figure S7 shows an example of the fitting procedure for SLs.

At 200 mW, the highest PCE value was obtained for the SL-CHOL sample at 18.4%, followed by HELs at 12.2%, SLs at 12.1% and free IR-780 at 7.6%. Cell membrane samples were not estimated due to photobleaching in this condition. PCE values were found to decrease with increasing laser power, a phenomenon observed before for inorganic nanoparticles [26]. A comparison with the literature is difficult since most estimations of PCE values were obtained from inorganic NPs [26,27,35,36]. Wang et al. found a PCE value of 16% for CuS NPs, and Tian et al. found 39% for Fe3O4 NPs, while Cole et al. investigated several types of Au nanostructures and found values between 30 and 55%. Due to plasmonic resonance, gold nanostructures can achieve PCE values close to 100% [37]. Organic dyes might also deteriorate with time, so estimations need to be carefully performed. Nevertheless, comparisons between samples prepared with the same batch are fair. From this analysis, one can conclude that synthetic liposomes are the best for PTT and that cholesterol improves heat generation. The enhancement might be related to the IR-780 arrangement and the lipid bilayer phase.

The cell viability study showed that IR-780 has a chemotherapeutic effect even without laser irradiation. This corroborates other articles in the literature that found similar effects for other cell lines, such as MCF7, A549 and EAT [10,38]. The effect of DOX is well established in the literature on chemotherapy and immunotherapy, since the drug might induce immunogenic cell death [19]. Encapsulating DOX in SL-CHOL with IR-780 reduced IC50 by almost 3-fold. However, at high concentrations, for example, at 7.5 μg/mL, the cell viability of SL-CHOL is lower than that of SL-DOX. The differences between the samples might be related to different particle sizes. SL-CHOL had a mean diameter of 120 nm, while SL-DOX was 164 nm, but the latter had a large size dispersity. It is known that the wrapping time increases for larger particle sizes [39,40], so it might be possible that aggregates at higher particle concentrations result in distinct internalization times, reducing the amount of intracellular DOX. Anyway, the data allowed us to design the heat-triggered DOX release study.

In Figure 7, we show that cells irradiated with the highest laser power, but without nanoparticles, had no effect on cell viability because there was no temperature variation. Vesicles with low IR-780 content also do not depend on laser irradiation for the same reason. The DOX amount has a strong influence on cell viability, but again, if the amount of IR-780 is not high enough, laser irradiation alone does not significantly affect cell viability (see Figure 7). Only increasing the amount of IR-780 in a manner that increases the local temperature results in decreasing cell viability. Figure S5 shows the temperature profile during PTT for the control, SL-DOX with 0.1 μg/mL and SL-DOX with 7.5 μg/mL. The CEM43 calculated for SL-DOX with the higher concentration was 6.5 min, while for the other samples, there was no detectable thermal dose. A huge decrease in cell survival to almost null after PTT at the highest IR-780 concentration was found. This effect arises from two main mechanisms, heat delivery and heat-triggered drug release, which increase the intracellular amount of DOX due to the therapeutic procedure. The synergetic effect (DOX plus heat) has been reported before, although the mechanism of cell death is still under debate and might not only be related to an increase in the intracellular DOX concentration [41].

As discussed before, some studies have used inorganic nanoparticles coated with cell membranes for photothermal-triggered DOX release. Sun et al. used gold nanocages, while Wang et al. worked with hollow copper sulfide [27,29]. The use of such nanostructures has advantages in comparison with organic molecules such as IR-780 since they do not show photobleaching and have good photostability. Despite the promise of gold nanostructures, they have not been approved for the clinic, although clinical studies have started [1]. Furthermore, IR-780 has a chemotherapeutic effect and can enhance cell death through ROS production (under laser irradiation), and it can be observed using near-infrared imaging. In the future, the fluorescence molecular tomography technique might be useful for breast cancer detection, indicating that near-infrared nanoparticles might have more clinical applications [9]. Potential future work could be the development of colon-targeted mucoadhesive nanoparticles containing IR-780 [42] for enhancing drug delivery through a photo-mediated response. Overall, the discussion above suggests that it might be interesting to evaluate the introduction of inorganic nanoparticles together with hybrid liposomes since the membrane functionality might still be active. In our opinion, iron oxide nanoparticles might be a natural choice since there are several products already approved for anemia, MRI contrast agents and thermal nanomedicine [1]. Indeed, this type of inorganic material is also relevant for photothermal therapy, magnetic nanoparticle hyperthermia, alternating current biosusceptometry and magnetic particle imaging [11,43,44]. So, we plan to explore a new multifunctional biomimetic nanoparticle for enhancing biomedical applications, including inorganic nanostructures combined with heat-triggered drug release.

## 5. Conclusions

The systematic comparison performed in this work supports the strong modulation of the photophysical and photothermal properties of IR-780 iodide by the microenvironment in which this dye is inserted. Specifically, we verified that in comparison with IR-780 solutions, both the fluorescent intensity and photoluminescence of low IR-780 amounts are enhanced when the dye is incorporated into synthetic lipid bilayers, mainly in the presence of moderate cholesterol content. In contrast, our results support that the presence of intrinsic proteins in cell-membrane-derived liposomes leads to a decrease in the IR-780 fluorescence. Furthermore, synthetic lipid membranes showed superior performance in diminishing IR-780 photobleaching when compared with cell-derived and hybrid membranes. Similar conclusions can be made concerning the photothermal conversion efficiency and the capability of each liposome to achieve the therapeutic thermal dose. In addition, we showed the heat-triggered doxorubicin release activated by IR-780 structured in synthetic lipid bilayers. So, we were able to show that although IR-780 has been claimed to be a multifunctional molecule, its expected photophysical and photothermal performance must be adjusted within the nanocarrier for enhanced biomedical applications.

## Figures and Tables

**Table 1:** Physical characteristics of the nanoparticles.

	Nanoparticle	Additional Information	Particle Size [nm]	Particle Concentration [liposome/mL]	Zeta Potential [mV]	2A_||_ [G]	IR-780 Concentration [mg/mL]
	Free IR-780	Ethanol	N/A	N/A	N/A		0.02–0.15
Synthetic liposomes	Phosphatidylcholine		119 ± 31	(1.0 ± 0.1) × 10^14^	−8 ± 2	50.2 ± 0.2 ^a^	N/A
						50.8 ± 0.2 ^a^	0.15
		Cholesterol	120 ± 34	(1.0 ± 0.1) × 10^14^	−12 ± 3	51.3 ± 0.1 ^b^	N/A
						51.9 ± 0.1 ^b^	0.15
		Doxorubicin	164 ± 60	(1.3 ± 0.3) × 10^14^	−22 ± 4	52.9 ± 0.2	0.15
Membrane vesicles	Erythrocyte		178 ± 74	(1.4 ± 0.1) × 10^12^	−24 ± 14	56.0 ± 0.5 ^c^	N/A
						*	0.08
						56.8 ± 0.5 ^c^	0.15
	Melanoma		198 ± 88	(4.8 ± 0.4) × 10^11^	−14 ± 2	53.0 ± 0.2 ^d^	N/A
						53.7 ± 0.5 ^d^	0.15
Hybrid liposomes	Erythrocyte		197 ± 88	(9.0 ± 0.3) × 10^12^	−26 ± 15	51.9 ± 0.1	0.15
	Melanoma		232 ± 130	(1.5 ± 0.1) × 10^12^	−6 ± 1	52.0 ± 0.1	0.15

* ESR characterization was not performed for EL sample containing 0.08 mg/mL IR-780. ^a^, ^b^, ^c^, and ^d^ are statistically different at *p* < 0.05 (*t*-test).

## Data Availability

Not applicable.

## Supplementary Materials

The following supporting information is available online at https://www.mdpi.com/article/10.3390/pharmaceutics15020444/s1. Figure S1: Nanoparticle size distribution curves; Figure S2: Nanoparticle zeta potential; Figure S3: Maximum temperature during PTT; Figure S4: Cell viability assay; Figure S5: Thermal dose; Figure S6: ESR nanoparticle characterization; Figure S7: Fitting procedure for PCE estimation; Table S1: Photothermal nanoparticle characterization.
