# Supplementary material for: Photothermal Properties of IR-780-Based Nanoparticles Depend on Nanocarrier Design: A Comparative Study on Synthetic Liposomes and Cell Membrane and Hybrid Biomimetic Vesicles
Source: Pharmaceutics. 2023 Jan 29;15(2):444. doi: 10.3390/pharmaceutics15020444 (PMC9961772; doi:10.3390/pharmaceutics15020444)
Supplement: Supplementary file 1 [file pharmaceutics-15-00444-s001.zip › pharmaceutics-2161491-supplementary.pdf]

Supplementary Material

Photothermal Properties of IR-780-Based Nanoparticles Depend on Nanocarrier Design: A Comparative Study on Synthetic Liposomes and Cell Membrane and Hybrid Biomimetic Vesicles

Júlia Muniz Barcelos[1], Tácio Gonçalves Hayasaki[2], Ricardo Costa de Santana[1], Eliana Martins Lima[2,3], Sebastião Antonio Mendanha[1,2,3] and Andris Figueiroa Bakuzis[1,3,*]

1 Institute of Physics, Federal University of Goiás, Goiânia 74690-900, GO, Brazil;
2 Farmatec, School of Pharmacy, Federal University of Goiás, Goiânia 74690-631, GO, Brazil;
3 CNanomed, Federal University of Goiás, Goiânia 74690-631, GO, Brazil;
*Correspondence: bakuzis@ufg.br;

Nanoparticle size distribution curves

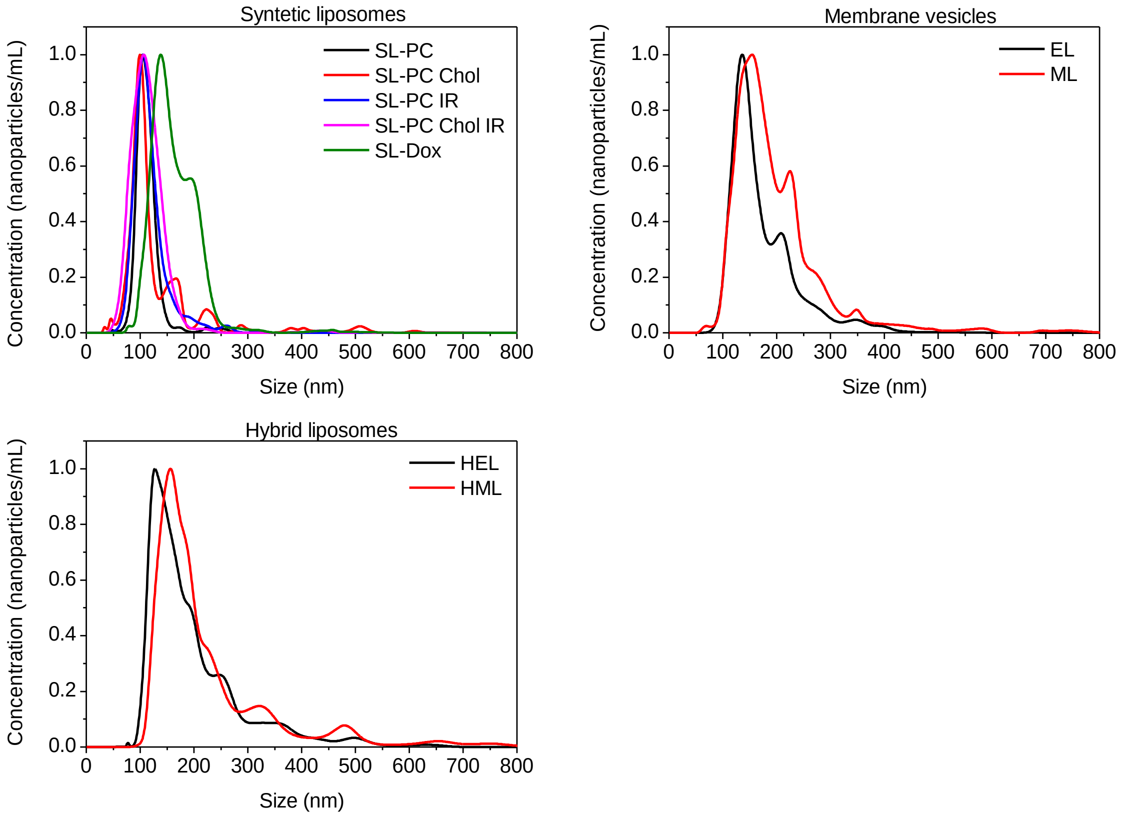

Figura S1 - Size distribution curves, obtained via nanotracking analysis (NTA) for the different types of synthetic liposomes (SL), membrane vesicles (MV) and hybrid liposomes (HL). From each curve was extracted a nanoparticle diameter expressed as (mean ± SD) in Table 1. DOX was loaded in a SL containing 40 mol% of cholesterol and 0.15 mg/mL of IR-780 was incorporated in SL, MV and HL samples.

Nanoparticle zeta potential

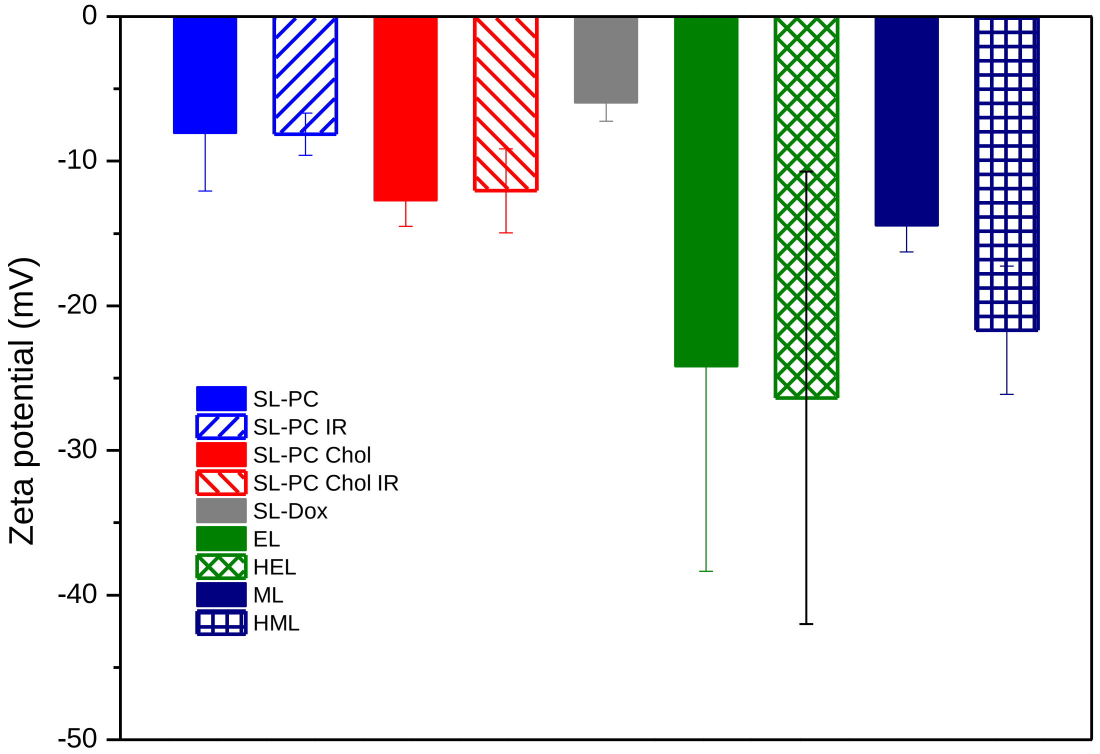

Figura S2 – Comparison of nanoparticles zeta potential values that are indicated in Table 1. The presence of IR-780 had not altered the liposome superficial charge.

Maximum temperature during PTT

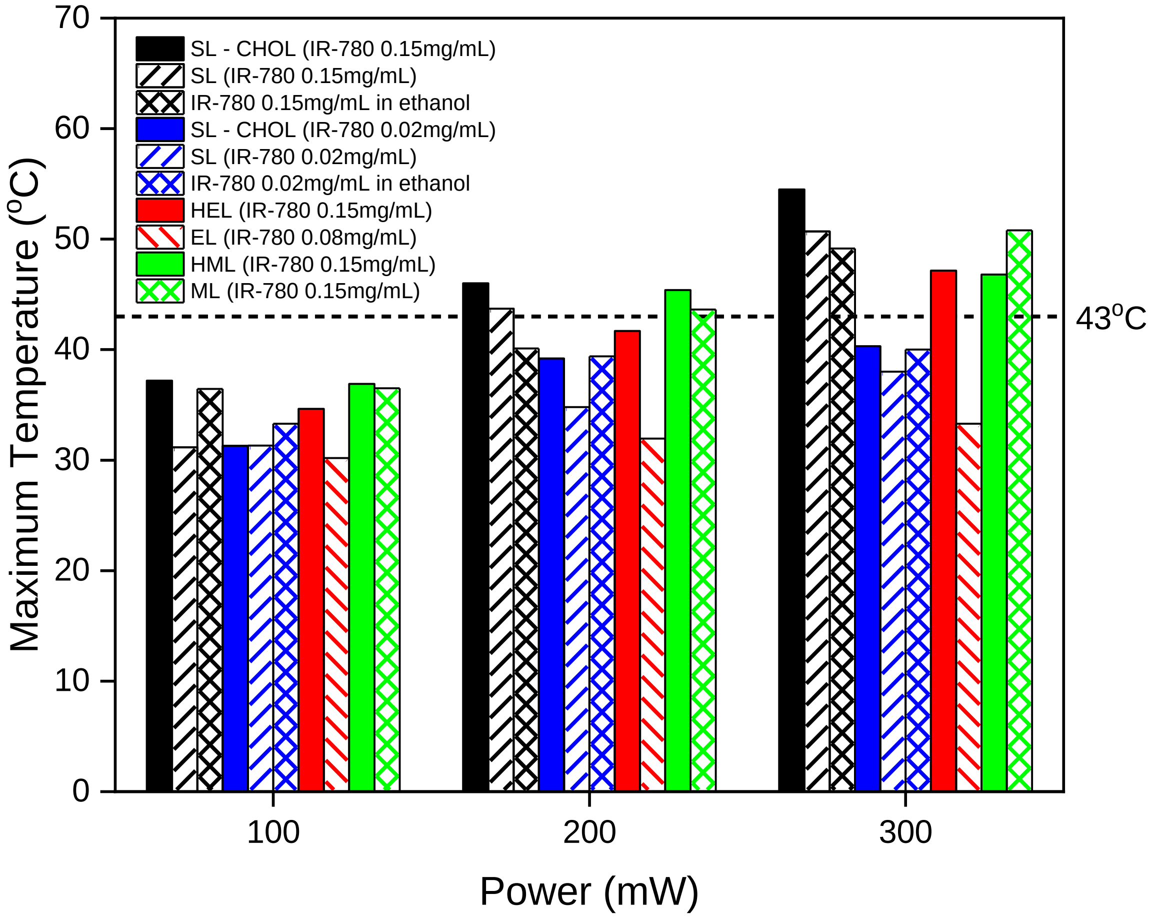

Figura S3 – Comparison of the maximum temperature achieved by each nanoparticle exposed to a diode laser (808 nm) as a function of it power. The photothermal characteristics of each nanoparticle are summarized in Table S1.

Photothermal nanoparticle characterization

Nanoparticle		IR-780 content [mg/mL]	Photothermal efficiency coefficient [%]			Environment temperature [°C]			Maximum temperature [°C]			ΔT [°C]		
			Laser power [mW]			Laser power [mW]			Laser power [mW]			Laser power [mW]		
			100	200	300	100	200	300	100	200	300	100	200	300
	Free IR-780	0.15	10 ± 1	7 ± 2	PBL	27	27	27	36	40	49	9	13	22
		0.02	3 ± 1	7 ± 1	PBL	27	27	27	33	39	40	6	12	13
synthetic liposomes	PC	0.15	15 ± 1	12 ± 1	3 ± 2	27	27	28	31	44	51	4	17	24
		0.02	PBL	PBL	PBL	26	28	28	31	35	38	5	7	10
	PC-CHOL	0.15	27 ± 1	18 ± 1	12 ± 3	27	27	27	37	46	55	10	19	28
		0.02	PBL	PBL	PBL	26	27	28	37	40	49	11	13	21
membrane vesicles	erythrocyte	0.08	PBL	PBL	PBL	26	27	27	30	32	33	4	5	6
	melanoma	0.03	PBL	PBL	PBL	27	28	27	37	44	51	10	16	24
hybrid liposomes	erythrocyte	0.15	21 ± 1	12 ± 1	PBL	27	28	27	35	42	47	8	14	20
	melanoma	0.15	29 ± 1	PBL	PBL	27	28	26	37	45	47	10	17	21

Table S1 – Comparison of nanoparticles photothermal proprieties with free IR-780 ethanolic solutions.

Cell viability assay

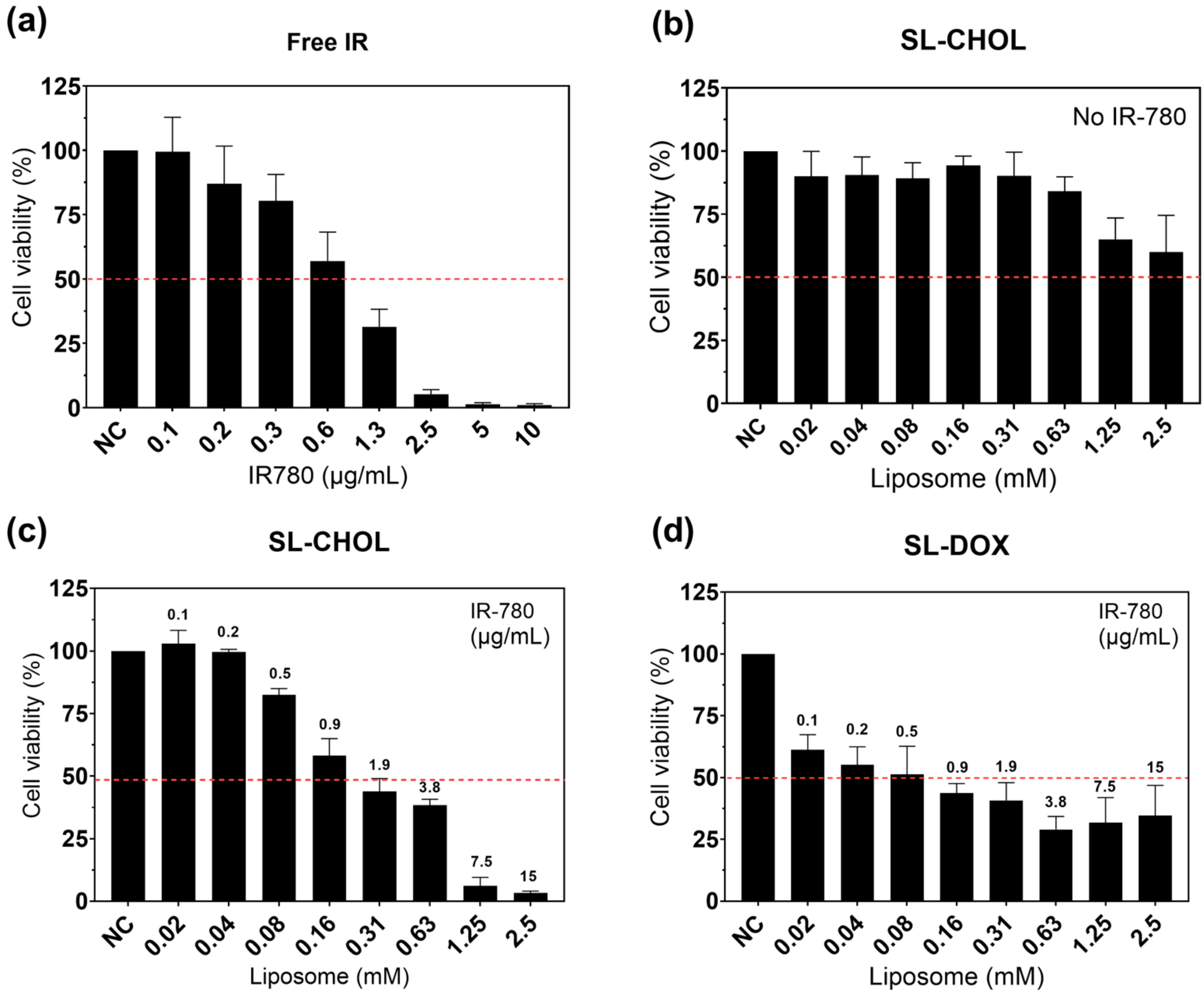

Figura S4 - Cell viability study without laser irradiation: (**a**) MTT study as function of free IR-780 concentration; (**b**) MTT investigation of the toxicity of SL-CHOL without IR-780; (**c**) MTT study of SL-CHOL containing IR-780; (**d**) MTT study of SL-DOX. DOX encapsulated in the vesicles decreases the IC_{50}. The variation of liposome concentration arises from dilution of the original sample. The IR-780 concentration is indicated for each liposome concentration.

Thermal dose

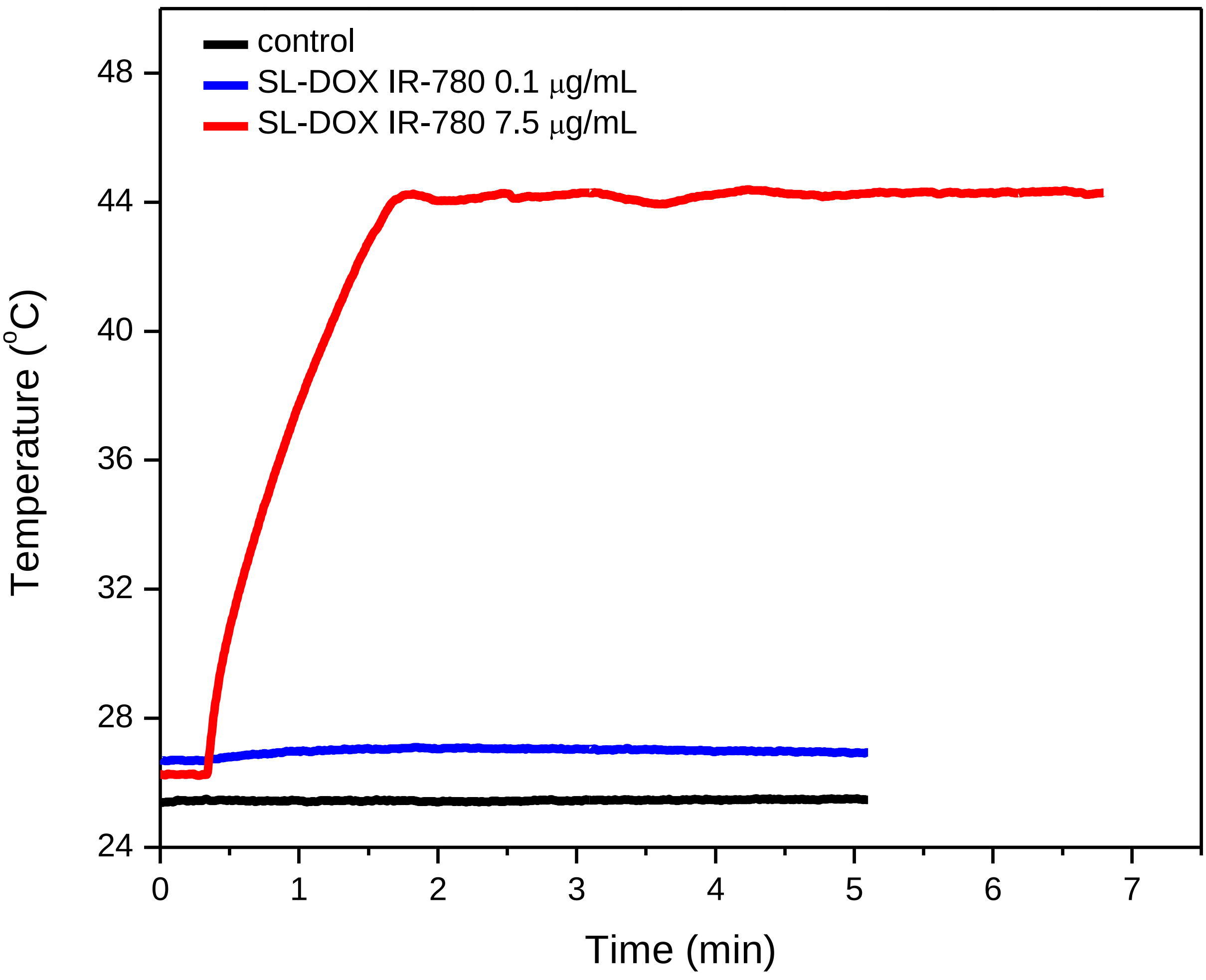

Figura S5 – Thermal dose applied for control sample (B16-F10 cell in culture medium) and for cells exposed to 0.1 and 7.5 ug/mL of SL-DOX nanoparticles.

ESR nanoparticle characterization

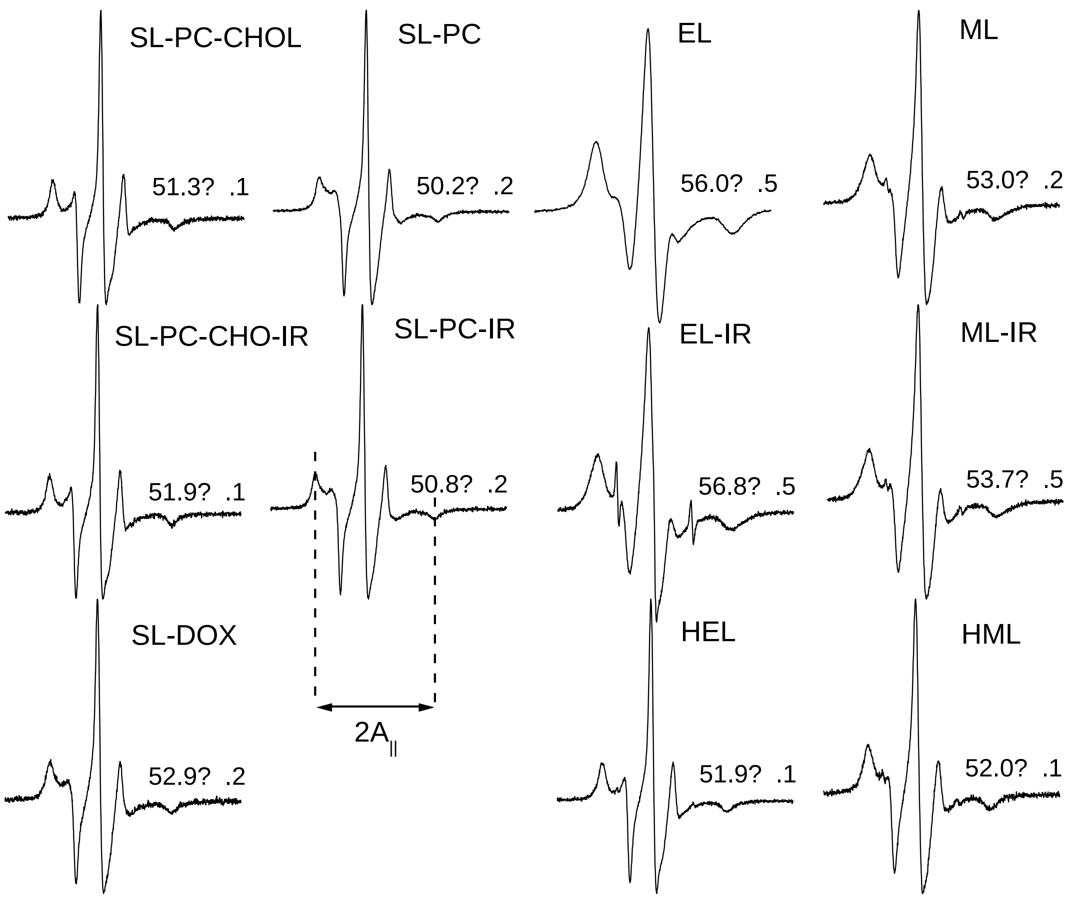

Figura S6 - ESR spectra of 5-DSA incorporated into the lipid bilayer of the synthetic liposomes (SL), membrane vesicles (MV) and hybrid liposomes (HL), including samples without IR-780 incorporation The variations in the EPR parameter $2A_{||}$ (outer hyperfine splitting) are also indicated, and this value is given by the magnetic field separation between the first peak and the last inverted peak of each spectrum. All ESR spectra was recorded using 100 G (X axis) as total scan range of the magnetic field and had their intensity expressed in arbitrary units (Y axis). The decrease in membrane fluidity promoted by cholesterol and/or IR-780 content may be evaluated as an increase in $2A_{||}$ values.

Fitting procedure for PCE estimation

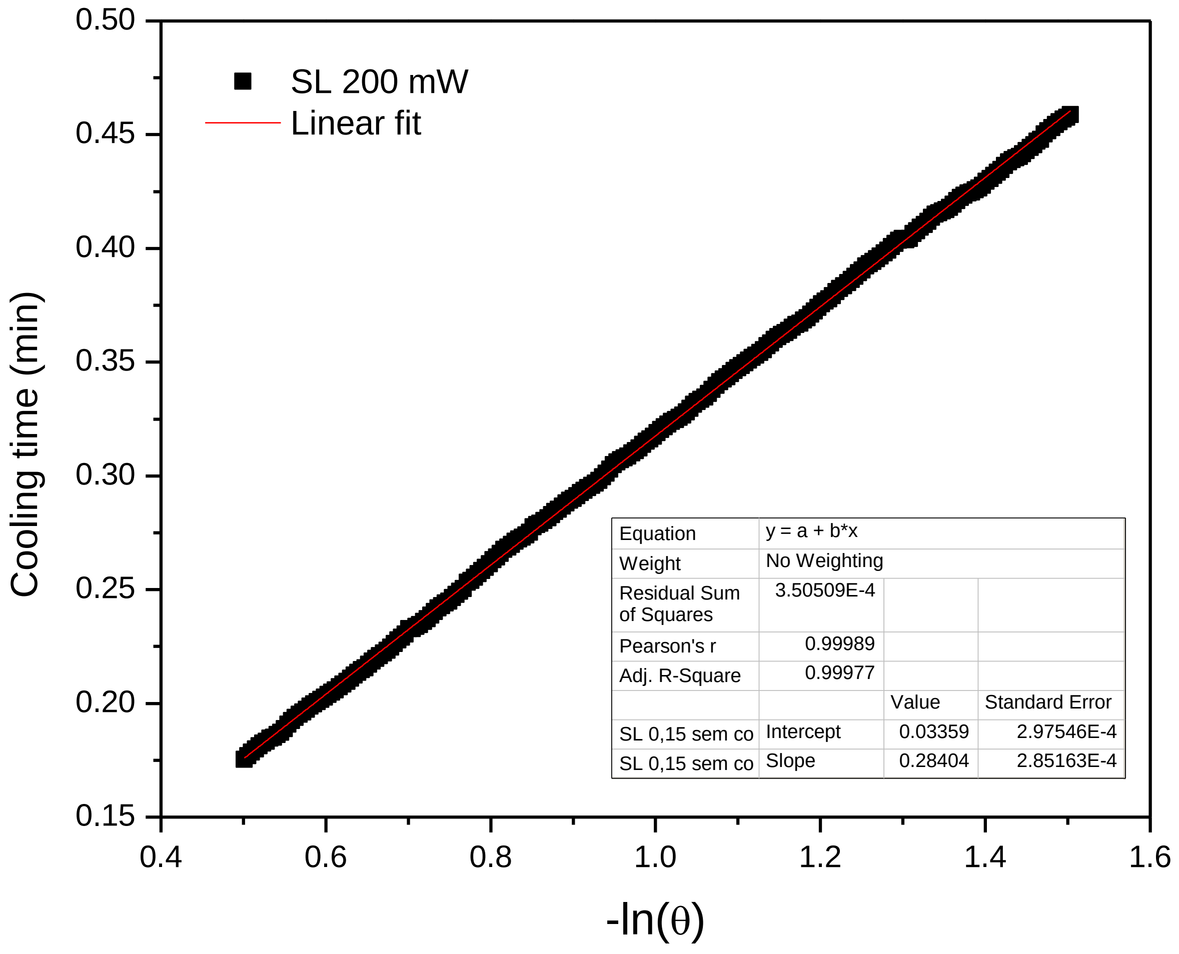

Figura S7 – Representative example of the fitting procedure performed to obtain PCE values summarized in Table S1.
